# Blow Flies Were One of the Possible Candidates for Transmission of Highly Pathogenic H5N1 Avian Influenza Virus during the 2004 Outbreaks in Japan

**DOI:** 10.1155/2011/652652

**Published:** 2010-12-28

**Authors:** Kyoko Sawabe, Keita Hoshino, Haruhiko Isawa, Toshinori Sasaki, Kyeong Soon Kim, Toshihiko Hayashi, Yoshio Tsuda, Hiromu Kurahashi, Mutsuo Kobayashi

**Affiliations:** Department of Medical Entomology, National Institute of Infectious Diseases, 1-23-1 Toyama, Shinjuku-ku, Tokyo 162-8640, Japan

## Abstract

The 2003-2004 H5N1 highly pathogenic avian influenza (HPAI) outbreaks in Japan were the first such outbreaks in 79 years in Japan. Epidemic outbreaks have been occurring in Southeast Asia, with the most recent in 2010. Knowledge of the transmission route responsible for the HPAI outbreaks in these countries remains elusive. Our studies strongly suggested that field and laboratory studies focusing on mechanical transmission by blow flies should be considered to control H5N1 avian influenza outbreaks, in particular in epidemic areas, where there are high densities of different fly species throughout the year. In this paper, we review these field and laboratory entomological studies and discuss the possibility of blow flies transmitting H5N1 viruses.

## 1. Avian Influenza Outbreaks in Japan

The H5N1 subtype of highly pathogenic avian influenza (HPAI) A virus has frequently infected wild and domestic ducks in Asia, causing huge economic damage to both poultry farms and governments in the affected countries. Most avian influenza viruses do not infect humans, but the 1997 outbreak of the H5N1 virus in Hong Kong [1, 2] alerted the medical community that some subtypes of avian influenza viruses include highly pathogenic strains that can affect humans. In this influenza virus outbreak, there were 6 deaths in the 18 human cases caused by the H5N1 subtype [3]. As of August 2, 2010, WHO has identified 502 human cases of H5N1 influenza around the world, and 298 of these were fatal [4]. In particular, H5N1 outbreaks have occurred recently in Egypt, Indonesia, and Vietnam. Therefore, H5N1 influenza virus can cause serious public health problems in birds and humans and is one of the most infectious avian diseases transmissible to humans.

**Figure 1:**
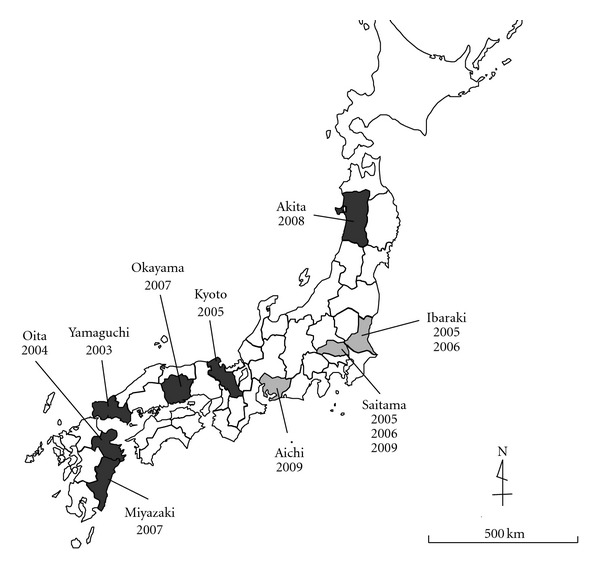
The avian influenza outbreaks occurred in Japan since 2003 reported by prefecture. Darker shading prefectures indicate the outbreak caused by H5N1 subtype of the virus during 2003–2007 and the detection of this subtype from wild birds in 2008. Lighter shading prefectures indicate the outbreaks caused by the other subtypes of the influenza viruses. This map shows prefectures and years when avian influenza outbreaks occurred. H5N2 subtype outbreaks were reported in Ibaraki and Saitama Prefectures in 2005 and 2006. The H3 subtype was reported in Saitama Prefecture in 2009 and the H7 subtype in Aichi Prefecture in 2009.

From January 2004 to March 2004, there were outbreaks of acute, highly transmissible, lethal diseases in chickens at four poultry farms in Japan: one in Oita, one in Yamaguchi, and two in Kyoto Prefecture (Figure 1). Virus isolates from infected chickens were all identified as influenza A virus of the H5N1 subtype [5]. Such highly pathogenic avian influenza (HPAI) epidemics had not been reported in Japan for 79 years. Two avian influenza outbreaks at poultry farms in Tamba Town, Kyoto Prefecture, were the last two outbreaks of the 2004 avian epidemics in Japan. Since then, there were outbreaks of H5N1 avian influenza in Okayama and Miyazaki Prefectures in 2007. The H5N1 virus was also isolated from dead Whooper swans, *Cygnus cygnus*, in 2008 in Towada Lake, Akita Prefecture [6]. In addition, outbreaks of other subtypes of avian influenza virus have frequently occurred in Japan. The H5N2 avian influenza was reported in Ibaraki and Saitama Prefectures in 2005 and 2006, the H3 subtype was reported in Saitama Prefecture in 2009, and the H7 subtype was in Aichi Prefecture in 2009. We know of no report suggesting that H5N1 virus could be transmitted efficaciously from person to person, but the possibility remains that such transmission could evolve [7]. 

## 2. The 2004 HPAI Outbreaks in Kyoto

**Figure 2:**
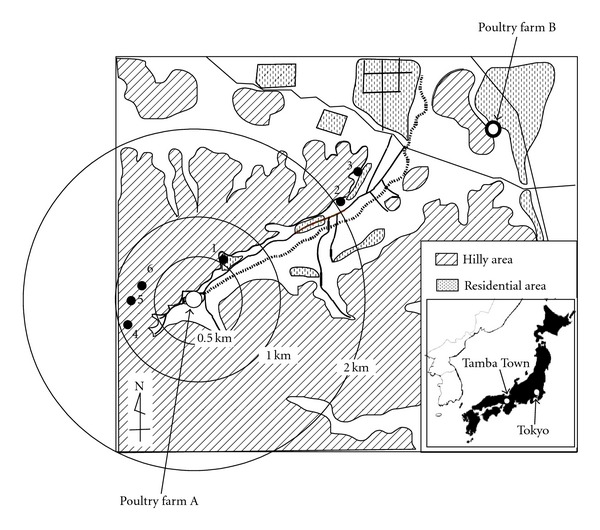
Sites in Tamba Town from which flies used in this study were collected. Site 1 is located at 600 m east from the poultry farm A. Sites 2 and 3 are located at over 2 km east from this farm and intermediate between two poultry farms, A and B. Sites 4–6 are located within 700–900 m west from the poultry farm A (redrawn from [11]).

Tamba Town (35°9′42′′ N and 135°26′31′′ E) is located in a hilly area 150–300 m above sea level, 50 km northwest of Kyoto City, Japan (Figure 2). Poultry farm A, a commercial layer chicken farm, had an outbreak of HPAI caused by H5N1 virus in February 2004, which was the most severe outbreak of the 2003-2004 epidemics. This farm is at the end of a small valley, with the flat valley basin used for rice cultivation and the hillside used as a plantation for coniferous trees. This outbreak resulted in the loss of 225,000 chickens due to infection and cure to try to control the outbreak. After the outbreak at poultry farm A, there was an outbreak at poultry farm B, a commercial broiler chicken farm 4 km northeast of poultry farm A. This outbreak started in early March 2004 and resulted in the loss of 15,000 chickens.

From the beginning of March 2004, studies targeting wild birds were undertaken to clarify the transmission route of H5N1 avian influenza virus in Tamba Town. During and after the outbreaks, virus surveillance was carried out for migrating birds around the outbreak areas in Kyoto. Although H5N1 viruses were isolated from seven large-billed crows, *Corvus macrorhynchos*, in Kyoto (A/crow/Kyoto/53/2004) and two in Osaka (A/crow/Osaka/102/2004), no influenza virus was found in any other species of the 102 dead birds (21 species in 11 families) examined. However, the exact transmission route has not yet been clarified [8].

## 3. Transmission Route of the H5N1 Virus into Japan

Knowledge of the transmission route responsible for the HPAI outbreaks in Southeast and East Asian countries still remains elusive [5, 9]. Four hypotheses have been suggested for transmission of H5N1 in the HPAI outbreaks in Japan [6]: (1) H5N1-virus-infected chickens may have been imported from other countries, (2) materials (e.g., vehicles and egg containers) from infected area may have been used, (3) viruses may have been carried on clothes, boots, hands, and so forth, and (4) infected wild birds may have carried H5N1 virus into poultry farms to infect chickens. In particular, it has been suggested that migratory birds carried the viruses and subsequently infected domestic and/or wild ducks [9]. The origin of the H5N1 strain isolated from Kyoto (A/chicken/Kyoto/3/2004) was traced back to a virus isolated from wild ducks in Guangdong Province, China, in 1996 (A/goose/Guangdong/1/1996). This virus strain caused further H5N1 outbreaks in Shandong Province, China, in 2003, in Korea in December 2003, and the 2003-2004 H5N1 HPAI outbreaks in Japan. Therefore, it is very likely that the 2004 epidemics H5N1 virus was transmitted to Japan from East Asian countries. 

The important question is how could the H5N1 virus be transmitted from virus-positive migratory wild birds to domestic poultry in Japan? We have noted that it is unlikely that wild birds directly transmitted influenza viruses to poultry in Japan, because all of the Japanese poultry farms, where H5N1 virus outbreaks occurred, had fowling nets in place to prevent entry of wild birds. However, flying insects (e.g., flies) can easily get through the nets and invade a poultry farm. We have shown that a chicken can eat all 31 blow flies put inside its cage in just 7 min [10]. A chicken can catch and break down the body of a fly and even catch and swallow a fly in flight. Therefore, we have been interested in whether, if a chicken eats blow flies carrying H5N1 virus, the chickens might become infected and develop symptoms of H5N1 influenza.

## 4. Did Blow Flies in Japan Transmit the H5N1 Virus?

**Figure 3:**
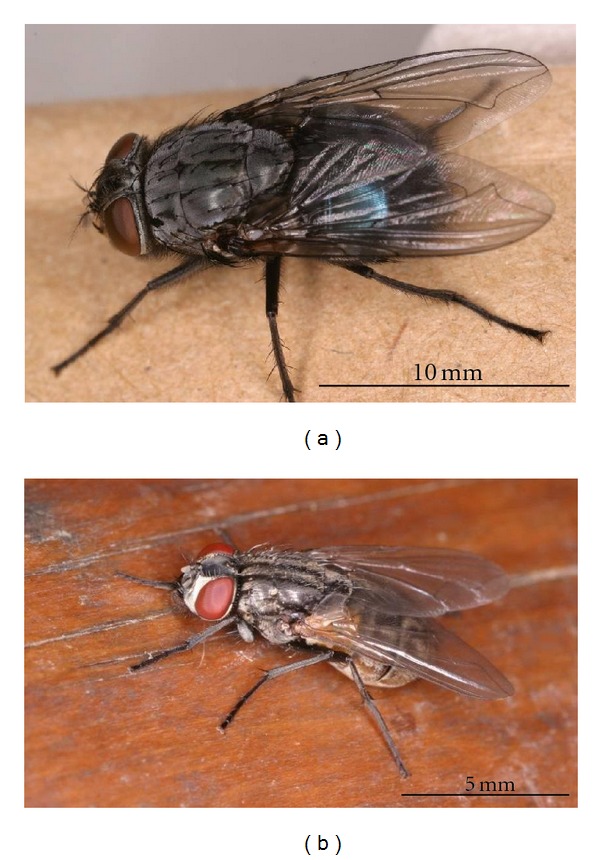
Blow fly *Calliphora nigribarbis *(a) and domestic house fly *Musca domestica* (b). These photos are available online at http://www.nih.go.jp/niid/entomology/pictures/pictures.html.

Although the spring season in Japan is cold, some fly species are present. However, no studies have been reported on the possible role of flies in transmission of H5N1 influenza virus. Therefore, an entomological survey was conducted in March 2004 to investigate the possibility of blow flies transmitting H5N1 virus, using flies collected from around the infected poultry farm in Tamba Town for virus detection and isolation. Blow fly collection was carried out on 10-11 March 2004, just after the H5N1 outbreak at poultry farm B [11]. A sunny place protected from strong wind was selected, and rotten fish bait was placed on the ground. A total of 926 flies were collected within a 2.3 km radius of poultry farm A in Tamba Town (Figure 2), representing eight fly species with >80% of the collected flies identified as either *Calliphora nigribarbis *Vollenhoven or *Aldrichina grahami *(Aldrich) (Figure 3). 

Influenza A virus matrix protein (M) and hemagglutinin (HA) genes were detected in the intestinal organs, crop, and gut of *C. nigribarbis *and *A. grahami* by reverse transcription-polymerase chain reaction (RT-PCR) [11]. The prevalence of H5 subtype virus (20–30%) was higher in flies of both species collected 600–700 m from poultry farm A and lower (10%) in flies collected >2 km from poultry farm A. We found that nearly 5% of *C. nigribarbis* collected around the affected areas contained infectious H5N1 viruses. Using RT-PCR with HA and M gene primers, 44 of 180 (24.4%) blow flies examined were identified as virus gene positive. Influenza viruses were isolated in embryonated chicken eggs from the intestinal organs of 2 of 10 blow flies (20%). The viral M, HA, and NA genes were amplified by PCR with universal primers, full-length sequences were analyzed, and sizes were found to be 991, 1,707, and 1,362 bp, respectively. These sequences showed high similarity to those of strains from chickens (A/chicken/Kyoto/3/2004) and crows (A/crows/Kyoto/53/2004) isolated during the 2004 outbreaks in Kyoto, with >99.9% identity for all three genes. The virus from *C. nigribarbis *(A/blow fly/Kyoto/93/2004) was characterized as an H5N1 subtype influenza A virus based on neuraminidase gene (NA) sequences. In addition, the HA1-HA2 connecting peptide sequence in the HA gene segment was RERRRKKR↓G. Finally, virus isolated from *C. nigribarbis *was characterized as a highly pathogenic H5N1 subtype influenza A virus.

## 5. H5N1 Virus Survival in Blow Flies

To investigate whether H5N1 virus could survive in the blow fly *C. nigribarbis*, we monitored the titer of infectious virus in flies after they were exposed to the H5N1 avian influenza A virus (A/duck/Hyogo/35/2001) [10]. Fifty female blow flies (Kyoto strain *C. nigribarbis*), approximately 14 days old, were put into a 20 cm^3^ fly cage for 3 h at 20°C with a piece of cotton impregnated with 10^8^ EID_50_/mL allantoic fluid from an H5N1 virus (A/duck/Hyogo/35/2001 [10])—infected egg diluted with MEM diluents [11]. Following the 3 h virus exposure, the blow flies were individually reared at 20°C or 10°C until tested. Incubation at 10°C was chosen because the average temperature around Tamba Town was 3.6°C in February and 6.4°C in March, with average daytime highs of 11.1°C in February and 13.1°C in March [12].

Crops and intestines dissected from flies' bodies at various times after virus exposure were used for virus isolation and titration. Virus was isolated from fly crops and intestines up to 24 h after exposure and from feces and vomit matter of 1 of 3 blow flies at 48 h after exposure (Table 1). At 14 d after exposure, no virus was isolated from any blow fly at 20°C or 10°C. The H5N1 viral gene could be detected in blow flies up to 14 d after exposure, although no viable virus was detected after 48 h after exposure. Almost all virus-positive samples had a titer of infectious H5N1 virus ranging from 0.5 to 4.63 TCID_50_, but 10 virus-positive samples had no detectable titer (<0.50 TCID_50_). The viable titer of H5N1 virus in the cotton put in the fly cages up to 48 h after exposure ranged from 4.50 to 5.00 TCID_50_.

## 6. Characteristics of the Blow Fly

Two species of the blow fly, *C. nigribarbis *and *A. graham*, are categorized as larger-sized fly species in particular in comparison to the house fly *Musca domestica* (L.); its body size is 5–8 mm (Figure 2). The body length of female *C. nigribarbis* is 11–15 mm and approximately 1.5 times larger than that of female *A. grahami *(8–13 mm). The capacity of the crop of female *C. nigribarbis* (average = 23 mL) is approximately five times greater than that of female *A. grahami *(average = 4.4 mL) [11]. The consumption rate of both *C. nigribarbis *and *A. grahami *might have been high because of their large body size. In fact, virus genes were found more often in *C. nigribarbis *than in *A. grahami* [11]. Stable flies, *Muscina stabulans *(Falle'n) and *M. angustifrons* (Loew), collected at the same collection sites and the same time as the fly surveillance in Kyoto, showed much smaller body size than *C. nigribarbis *and *A. grahami*, and no virus was detected in these smaller-sized flies [11].

Blow flies prefer to lick animal carcasses and droppings. If food for blow flies is contaminated by pathogens, the flies might ingest significant numbers of pathogens. One possible mechanism for mechanical transmission of pathogens by blow flies is regurgitation and the feces on the food source [13, 14]. The effectiveness of mechanical transmission through regurgitation may depend on the viability and titer of pathogens in the fly's body. The accumulated droppings at a poultry farm should be a good breeding site for blow flies. If the flies reproduced at a poultry farm, they should have many opportunities for contact with viruses in the feces of infected chickens and/or their dead bodies.

## 7. Flight Capacity of *C. nigribarbis*

*Calliphora nigribarbis* has a characteristic temperate-zone life cycle. For example, in Japan, they become more active between winter and spring for migration and reproduction [15, 16]. It is well known that *C. nigribarbis* has excellent flight capacity and high dispersal ability. They have been identified by weather ships at stations located on the Pacific Ocean and East China Sea, 300–450 km from Kyushu Island, Japan [17]. It was also suggested that the number of flies found in autumn in the Kyushu District appears to increase due to their transoceanic migration [18]. Female blow flies can survive for about one year in Japan [17], in comparison to the house fly *Musca domestica* which has a mean longevity of 34.2 days [19]. The longevity and high dispersal ability of blow flies may also result in wide dispersion of viruses that they carry.

Mark-release-recapture experiments conducted at Tamba Town in 2005 suggested that *C. nigribarbis* generally could migrate up to 2-3 km in 24 h [20]. The distance between the two poultry farms in Kyoto prefecture, where the two H5N1 virus outbreaks took place in 2004, was approximately 4 km. In fact, 10% of all *C. nigribarbis *flies collected at a site intermediate between the affected farms expressed H5N1 virus genes [11]. Viable titers of H5N1 influenza virus, but not virus replication, were detected for up to 24 h in the crop and intestine of virus-exposed *C. nigribarbis *[10]. The presence of infectious virus in blow flies for 24 h could have a strong implication for virus dispersion since blow flies, with their excellent flight capacity, could transport the H5N1 virus over significant distances. In addition, H5N1 virus has been isolated from feces and vomit matter of blow flies at the 48 h postexposure, but virus titers in flies at 48 h were lower than that of the virus-containing cotton used in these experiments. This suggested that the viability of influenza virus decreases steadily in the blow fly crop and intestine, although some infectious virus remains for longer than 24 h. Therefore, *C. nigribarbis* could transport H5N1 virus to poultry farms 2-3 km apart.

## 8. Field Surveillance and H5 Influenza Virus Detection from Blow Flies

**Figure 4:**
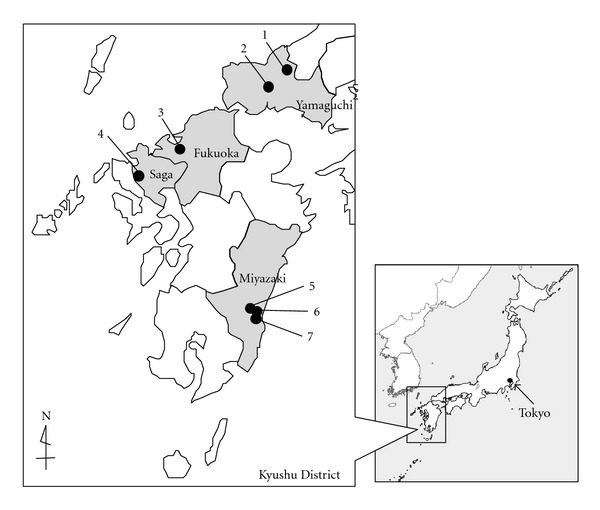
Location of blow fly collection sites (closed circles) in Kyushu District and Yamaguchi Prefecture during 2005–2007.

How often do H5 influenza viruses migrate to Japan? Which subtypes of H5 influenza virus migrate to Japan? To investigate these questions, we conducted surveillance for blow flies in the Kyushu District and Yamaguchi Prefecture during 2005–2007 to try to evaluate possible invasions of H5 influenza viruses (Figure 4). The collection sites in Yamaguchi and Miyazaki Prefectures were close to the affected poultry farms in which HPAI H5N1 outbreaks occurred in 2003 and 2007, respectively. The sites in Fukuoka and Nagasaki Prefectures locate transoceanic migration routes of bird and insect. A total of 1,887 blow flies were collected using rotten fish as bait. Crops and intestines dissected from 20 flies were pooled and tested for influenza virus HA and M gene fragments by RT-PCR with nested PCR, following previous studies [10, 11]. No H5 influenza virus gene was detected from a total number of 96 fly pools examined (Table 2). However, one of the 31 pools collected from Nishiarita Town, Saga Prefecture, in November 2006 was positive for the influenza virus M gene. Although the virus in this pool was not H5 subtype and its subtype has not been identified yet by sequence analysis, it was confirmed to be the influenza A virus. This result showed that blow flies have the capacity to ingest different influenza virus types and/or subtypes, probably from drops of migratory birds.

## 9. Domestic House Flies also Transmit Pathogens

It is well known that the domestic house fly, *Musca domestica* spp., and some other fly species can transmit many kinds of pathogens mechanically [21–25]. In particular, *M. domestica *spp. are the most important fly species at poultry farms [26] with regard to mechanical transmission of >30 different pathogens [13], for example, bacteria, protozoa, viruses, and parasite oocysts and eggs. Some viruses can be transported to animals by contact with contaminated body surfaces of flies. In the case of the house fly, it has been shown that rotavirus can be mechanically transported by contaminated fly surfaces [21]. House flies frequently defecate while feeding and resting on food surfaces [27]. However, in studies of *C. nigribarbis*, neither defecation nor vomiting was observed within 24 h after feeding (data not shown). The body surface of the house fly could be contaminated by viruses easier than that of blow flies. This would suggest that the mechanisms of virus transmission by blow flies could be different from those of house flies. Therefore, to evaluate virus transmission mechanisms that are more complex than contact with a contaminated fly surface, blow fly intestinal organs, crop, and gut were analyzed for their possible role in transmission of avian influenza virus. A seasonal consideration is that *M. domestica vicina *populations are generally highest in the summer in Japan. In fact, no house fly was found around any poultry farm or pigpen in Tamba Town during our survey in March. Therefore, it seems reasonable that winter blow flies may be involved in transmission of winter pathogens, like influenza virus, by maintaining minimum infectious titers.

## 10. Conclusion

We have suggested here that blow flies are likely candidates for mechanical transmission of HPAI because of their ecological and physiological characteristics as reviewed here. In fact, blow flies have already been recognized as important vectors for mechanical transmission of several serious infectious diseases, that is, poxvirus [28], rabbit hemorrhagic disease [29], and paratuberculosis [30]. Recently, it has been reported that the H5N1 viral gene was detected in house flies [31] and engorged mosquitoes [32]. We suggest that mechanical transmission by flies may also be involved in the outbreak and pandemic of infectious diseases other than HPAI. However, although there are high densities of a variety of fly species during all seasons in Southeast Asia, their ability to transmit viruses has not been evaluated. The prevalence of H5N1 avian influenza is still a public health problem for birds and humans. Therefore, field and laboratory studies on mechanical transmission of pathogens by flies would be very important for controlling H5N1 avian influenza outbreaks, at least in epidemic Southeast Asian countries.

Recently, the H5N1 virus surveillance conducted in Indonesia suggested that pigs are at risk of infection during outbreaks and pigs can serve as intermediate hosts in which this avian virus can adapt to mammals [33]. They also found the evidence of pig-to-pig transmission of this virus without any significant influenza-like signs. The transmission mechanism of this virus became more complicated and serious. As we introduced in the previous section, blow flies prefer to lick carcasses and droppings of not only chickens but also pigs. Furthermore, we can assume that the flies can access the pigpen easier than the poultry farm. This finding from Indonesia [33] strongly suggest that it is important to pay attention to pigpens as well as poultry farms within 2-3 km, where viable H5N1 viruses are transmitted by blow flies.

## Figures and Tables

**Table 1:** Diagnostic results of H5N1 influenza A viruses from crop, intestine, and feces and vomit matters of a blow fly after experimental exposure to the virus solution (from [10]).

Postexposure		Replicate	Crop			Intestine			Feces and vomit matters			Virus-exposed cotton		
			VI	TCID_50_	PCRs	VI	TCID_50_	PCRs	VI	TCID_50_	PCRs	VI	TCID_50_^a^	PCRs
20^*º*^C		1	+	3.50	+	+	3.50	+						
		2	+	3.33	+	+	4.00	+						
		3	+	1.83	+	+	0.50	+						
		4	+	4.60	+	+	3.50	+						
	3 h	5	+	2.60	+	+	3.00	+				NT	5.00	+
		6	+	1.67	+	+	1.67	+						
		7	+	<0.50	+	+	<0.50	+						
		8	+	4.63	+	+	3.50	+						
		9	+	3.00	+	+	3.63	+						
		10	+	<0.50	+	+	<0.50	+						
		1	+	<0.50	+	+	3.50	+	−	NT	+			
	6 h	2	+	<0.50	+	+	1.50	+	−	NT	+	NT	4.50	+
		3	−	<0.50	+	−	<0.50	+	−	NT	+			
		1	+	1.00	+	+	1.00	+	−	NT	+			
	9 h	2	+	<0.50	+	+	<0.50	+	−	NT	+	NT	5.60	+
		3	−	<0.50	+	−	<0.50	+	−	NT	+			
		1	−	<0.50	+	−	<0.50	+	−	NT	+			
	24 h	2	−	<0.50	+	−	<0.50	+	−	NT	+	NT	4.67	+
		3	+	1.67	+	+	<0.50	+	−	NT	+			
		1	−	NT	+	−	NT	+	+	<0.50	+			
	48 h	2	−	NT	+	−	NT	+	−	NT	+	NT	4.83	+
		3	−	NT	+	−	NT	+	−	NT	+			
		1	−	NT	+	−	NT	+	−	NT	+			
	14 d	2	−	NT	+	−	NT	+	−	NT	+	NT	<0.50	+
		3	−	NT	+	−	NT	+	−	NT	+			

		1	−	NT	+	−	NT	+	−	NT	+			
10^*º*^C	14 d	2	−	NT	+	−	NT	+	−	NT	+	NT	<0.50	+
		3	−	NT	+	−	NT	+	−	NT	+			

VI: virus isolation using embryonated chicken eggs, TCID_50_: virus titers (log_10_ TCID_50_/0.05 mL) were calculated by the inoculation onto MDCK cells,

PCRs: RT-PCR performed with specific primers for the HA and M genes and followed by a nested PCR with primers for the HA gene, NT: not tested.

^a^The average of two times of the virus titration.

**Table 2:** Detection of A/H5 influenza virus gene from blow flies, *Calliphora nigribarbis*, collected during 2004–2006.

Fly collection		No. tested	No. pools	No. positive fly pools^a^		
Sites*	Date			HA	HA nested	M
1. Ato Town, Yamaguchi	29. Oct. 2004	100	5	0	0	0
1. Ato Town, Yamaguchi	25. Oct. 2005	60	3	0	0	0
2. Yamaguchi City, Yamaguchi	30. Oct. 2005	18	1	0	0	0
3. Fukuoka City, Fukuoka	31. Oct. 2005	100	5	0	0	0
3. Fukuoka City, Fukuoka	28–30. Nov. 2006	340	17	0	0	0
**4. Nishiarita Town, Saga**	**28–30. Nov. 2006**	**620**	**31**	**0**	**0**	**1**
4. Nishiarita Town, Saga	7. Feb. 2007	319	16	0	0	0
5. Sadohara Town, Miyazaki	26. Jan. 2007	256	13	0	0	0
6. Kojo Town, Miyazaki	27. Jan. 2007	24	2	0	0	0
7. Kiyotake Town, Miyazaki	27. Jan. 2007	50	3	0	0	0

Total		1,887	96	0	0	1

^a^Crops and guts dissected from twenty flies were pooled and tested for the detection of HA and M gene fragments by using RT-PCR and following nested PCR.

*See Figure 4 for more information of each site.

## References

[1] Subbarao K, Klimov A, Katz J (1998). Characterization of an avian influenza A (H5N1) virus isolated from a child with a fatal respiratory illness. *Science*.

[2] Yuen KY, Chan PKS, Peiris M (1998). Clinical features and rapid viral diagnosis of human disease associated with avian influenza A H5N1 virus. *The Lancet*.

[3] Peiris JSM, Yu WC, Leung CW (2004). Re-emergence of fatal human influenza A subtype H5N1 disease. *The Lancet*.

[4] [WHO] World Health Organization Cumulative number of confirmed human cases of avian influenza A/(H5N1) reported to WHO. http://www.who.int/csr/disease/avian_influenza/country/cases_table_2010_07_29/en/index.html.

[5] Mase M, Tsukamoto K, Imada T (2005). Characterization of H5N1 influenza a viruses isolated during the 2003-2004 influenza outbreaks in Japan. *Virology*.

[6] [NIAH] National Institute of Animal Health Disease Information, Avian influenza. http://niah.naro.affrc.go.jp/disease/poultry/tori_influenza.html.

[7] Yang Y, Halloran ME, Sugimoto JD, Longini IM (2007). Detecting human-to-human transmission of avian influenza A (H5N1). *Emerging Infectious Diseases*.

[8] Food Safety and Consumer Bureau, Ministry of Agriculture, Forestry and Fisheries, Japan Report of highly pathogenic avian influenza infection route elucidation team. Routes of infection of highly pathogenic avian influenza in Japan. http://www.maff.go.jp/j/syouan/douei/tori/pdf/040630e_report.pdf.

[9] Lee YJ, Choi YK, Kim YJ (2008). Highly pathogenic avian influenza virus (H5N1) in domestic poultry and relationship with migratory birds, South Korea. *Emerging Infectious Diseases*.

[10] Sawabe K, Tanabayashi K, Hotta A (2009). Survival of avian H5N1 influenza a viruses in *Calliphora nigribarbis* (Diptera: Calliphoridae). *Journal of Medical Entomology*.

[11] Sawabe K, Hoshino K, Isawa H (2006). Detection and isolation of highly pathogenic H5N1 avian influenza A viruses from blow flies collected in the vicinity of an infected poultry farm in Kyoto, Japan, 2004. *The American Journal of Tropical Medicine and Hygiene*.

[12] Japan Meteorological Agency Weather, climate and earthquake information, 2004, Sonobe District data. http://www.data.jma.go.jp/obd/stats/etrn/index.php.

[13] Greenberg B (1973). *Flies and Disease: Vol. II. Biology and Disease Transmission*.

[14] Crosskey RW, Lane RP, Lane RP, Crosskey RW (1993). House-flies, blow-flies and their allies (Calyptrate: Diptera). *Medical Insects and Arachnids*.

[15] Kurahashi H, Kawai S, Shudo C, Wada Y (1994). The life history of *Calliphora nigribarbis* Vollenhoven in Mt. Hachijo-Fuji, Hachijo Island. *Japan Journal Sanitary and Zoology*.

[16] Kurahashi H (1979). Breeding of flies. *Insectarium*.

[17] Kurahashi H (1991). The calyptrate muscoid flies collected on weather ships located at the ocean weather stations. *Japan Journal of Sanitary and Zoology*.

[18] Kurahashi H, Suenaga O (1997). Witnessing hundreds of *Calliphora nigribarbis* in migratory flight and landing in Nagasaki, Western Japan. *Medical Entomology and Zoology*.

[19] Rockstein M (1957). Longevity of male and female house flies. *Journal of Gerontology*.

[20] Tsuda Y, Hayashi T, Higa Y (2009). Dispersal of a blow fly, *Calliphora nigribarbis*, in relation to the dissemination of highly pathogenic avian influenza virus. *Japanese Journal of Infectious Diseases*.

[21] Tan SW, Yap KL, Lee HL (1997). Mechanical transport of rotavirus by the legs and wings of *Musca domestica* (Diptera: Muscidae). *Journal of Medical Entomology*.

[22] Iwasa M, Makino SI, Asakura H, Kobori H, Morimoto Y (1999). Detection of *Escherichia coli* O157:H7 from *Musca domestica* (Diptera: Muscidae) at a cattle farm in Japan. *Journal of Medical Entomology*.

[23] Kobayashi M, Sasaki T, Saito N (1999). Houseflies: not simple mechanical vectors of enterohemorrhagic *Escherichia coli* O157:H7. *The American Journal of Tropical Medicine and Hygiene*.

[24] Sasaki T, Kobayashi M, Agui N (2000). Epidemiological potential of excretion and regurgitation by *Musca domestica* (Diptera: Muscidae) in the dissemination of *Escherichia coli* O157: H7 to food. *Journal of Medical Entomology*.

[25] Calibeo-Hayes D, Denning SS, Stringham SM, Guy JS, Smith LG, Watson DW (2003). Mechanical transmission of turkey coronavirus by domestic houseflies (*Musca domestica* Linnaeaus). *Avian Diseases*.

[26] Axtell RC (1999). Poultry integrated pest management: status and future. *Integrated Pest Management Reviews*.

[27] Hainsworth FR, Fisher G, Precup E (1990). Rates of energy processing by blowflies: the uses for a joule vary with food quality and quantity. *The Journal of Experimental Biology*.

[28] Docherty DE, Long RI, Flickinger EL, Locke LN (1991). Isolation of poxvirus from debilitating cutaneous lesions on four immature grackles (*Quiscalus* sp.). *Avian Diseases*.

[29] Asgari S, Hardy JRE, Sinclair RG, Cooke BD (1998). Field evidence for mechanical transmission of rabbit haemorrhagic disease virus (RHDV) by flies (Diptera: Calliphoridae) among wild rabbits in Australia. *Virus Research*.

[30] Fischer OA, Matlova L, Dvorska L (2004). Blowflies *Calliphora vicina* and *Lucilia sericata* as passive vectors of *Mycobacterium avium* subsp. *avium, M.a. paratuberculosis* and *M.a. horminissuis*. *Medical and Veterinary Entomology*.

[31] Sievert K, Alvarez R, Cortada R, Valks M (2006). House flies carrying avian influenza virus (AIV). *International Pest Control*.

[32] Barbazan P, Thitithanyanont A, Missé D (2008). Detection of H5N1 avian influenza virus from mosquitoes collected in an infected poultry farm in Thailand. *Vector-Borne and Zoonotic Diseases*.

[33] Nidom CA, Takano R, Yamada S (2010). Influenza a (H5N1) viruses from pigs, Indonesia. *Emerging Infectious Diseases*.

